# Plastic leachates impair growth and oxygen production in *Prochlorococcus*, the ocean’s most abundant photosynthetic bacteria

**DOI:** 10.1038/s42003-019-0410-x

**Published:** 2019-05-14

**Authors:** Sasha G. Tetu, Indrani Sarker, Verena Schrameyer, Russell Pickford, Liam D. H. Elbourne, Lisa R. Moore, Ian T. Paulsen

**Affiliations:** 1 Molecular Sciences, Macquarie University, Sydney, 2109 NSW Australia; 2 Marine Biological Section, Department of Biology, University of Copenhagen, Helsingør, 3100 Denmark; 3 Bioanalytical Mass Spectrometry Facility, University of New South Wales, Sydney, 2052 NSW Australia

**Keywords:** Environmental microbiology, Bacteria

## Abstract

Plastic pollution is a global threat to marine ecosystems. Plastic litter can leach a variety of substances into marine environments; however, virtually nothing is known regarding how this affects photosynthetic bacteria at the base of the marine food web. To address this, we investigated the effect of plastic leachate exposure on marine *Prochlorococcus*, widely considered the most abundant photosynthetic organism on Earth and vital contributors to global primary production and carbon cycling. Two strains of *Prochlorococcus* representing distinct ecotypes were exposed to leachate from common plastic items: high-density polyethylene bags and polyvinyl chloride matting. We show leachate exposure strongly impairs *Prochlorococcus* in vitro growth and photosynthetic capacity and results in genome-wide transcriptional changes. The strains showed distinct differences in the extent and timing of their response to each leachate. Consequently, plastic leachate exposure could influence marine *Prochlorococcus* community composition and potentially the broader composition and productivity of ocean phytoplankton communities.

## Abstract (web summary)

Sasha Tetu et al. have examined the effects of plastic leachate exposure on the marine bacteria *Prochlorococcus*, the most abundant photosynthetic organism on Earth. They find plastic leachates impair growth, photosynthetic capacity and cause global changes in transcription.

## Introduction

Plastic debris has been estimated to cause more than US$13 billion in economic damage to marine ecosystems each year and is now widely recognized to be a serious threat to the marine environment^1^. The current rate of increase in worldwide plastic production, poor end-of-life plastic waste management, and slow environmental degradation rates^2^ mean that further increases in the levels of marine plastic pollution are widely considered inevitable^3,4^.

Hazards associated with leaching of chemicals from marine plastics have received substantially less attention than issues linked to ingestion and entanglement^5^. Most engineered (non-biodegradable) plastic polymers are considered highly stable and biologically inert. However, various chemical compounds are added during manufacture of most plastic products to improve performance, aging and functionality and are generally not chemically bound to the plastic polymer^6^. Such substances, which can subsequently leach from plastics, include catalyst remnants, polymerization solvents, plasticizers, metals, dyes, flame-retardants, UV stabilizers, antioxidants and antimicrobials, detailed in the recent review by Hahaladakis and colleagues^6^. Many of these additives have been detected in marine and estuarine waters worldwide at concentrations ranging up to micrograms per litre^5^. Many plastics can also adsorb hydrophobic organic chemicals and may therefore also transport compounds derived from the surrounding environment^7^. Although the processes associated with delivery of various compounds to marine environments from plastic litter are currently poorly understood, a recent study on plastic ageing in marine water indicated that some polyvinyl chloride (PVC) additives are released following more than a year of immersion, due to degradation and progressive exposure of deeper layers of the material to the environment^8^.

Existing work on plastic leachate impacts has been largely limited to toxicological studies on model zooplankton. For example, toxicity of plastic leachates has been demonstrated for crustaceans (*Daphnia magna*^9^ and *Nitroca sinipes*^10^), barnacle larvae (*Amphibalanus amphitrite*)^11^ and mussel embryos (*Perna perna*)^12^. These studies have shown that leachates from plastic pollution represent a potential risk to some marine eukaryotic organisms whilst highlighting the high degree of variability in toxicity of leachates from different plastic items^5^. Understanding of potential effects of plastic leachates on marine microorganisms lags even further behind. The only study to date relating to plastic leachate effects on marine bacteria reported short-term growth stimulation of marine heterotrophic bacteria via supply of dissolved organic carbon^13^ and does not address effects on marine primary producers.

Bacteria of the cyanobacterial genus *Prochlorococcus* are the most numerous photosynthetic cells in the ocean^14^, with an estimated mean global population of ~10^27^ cells and primary productivity of 4 Gt C y^−1^ in some oligotrophic ocean regions^15^. Members of this genus are divided into two physiologically and phylogenetically distinct groups, high-light (HL) and low-light (LL) adapted clades, with HL representatives tending to be more abundant at shallower depths^16^. The role of *Prochlorococcus* in global oxygen production, carbon fixation and biogeochemical cycling indicate that they are important organisms to consider in determining the potential effects of stressors on marine ecosystems. Previous work has indicated that *Prochlorococcus* may be particularly sensitive to organic pollutants and other environmental stressors including UV radiation and elevated copper concentrations^17–21^.

Here we examined the response of two *Prochlorococcus* strains, MIT9312 (HLII Clade) and NATL2A (LLI Clade), two representative ecotypes found throughout tropical and subtropical oceans, to in vitro exposure to leachates from common plastic products. Experiments were performed using common plastic items composed of high-density polyethylene (HDPE) and PVC, two of the most commonly produced polymer types. We report growth, photosynthetic and transcriptomic effects that indicate plastic leachates have the potential to deleteriously affect marine phototrophic bacterial communities, with possible consequences for ocean primary productivity.

## Results

### Plastic leachates negatively affect *Prochlorococcus* growth

Leachates of common plastic items (HDPE shopping bags and PVC matting) were made using a base of sterile artificial seawater. Exponential phase cultures of *Prochlorococcus* were inoculated into media containing a range of leachate dilutions (generated by volume/volume dilutions in AMP1 media of 50, 25, 12.5, 6.25 and 3.125% for HDPE; 10, 2, 1, 0.5 and 0.25% for PVC) alongside equivalent controls (AMP1 media with no leachate addition). The leachate dilutions used for growth and photophysiology monitoring were chosen following preliminary tests that indicated PVC leachate had a considerably greater effect than HDPE at equivalent concentrations.

**Fig. 1:**
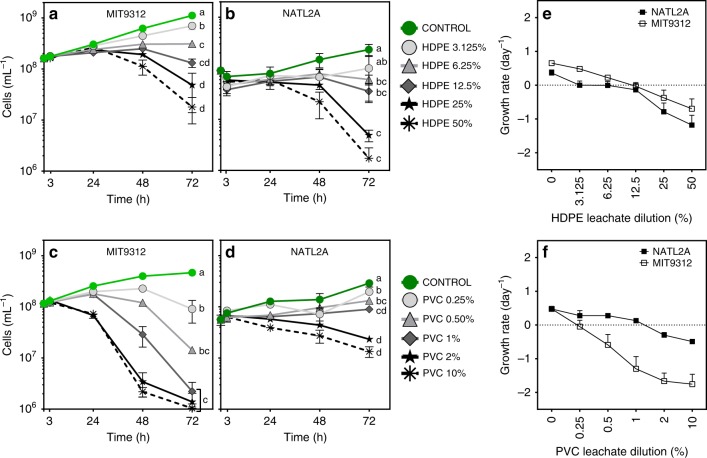
Population growth of *Prochlorococcus* MIT9312 and NATL2A in the presence of diluted HDPE and PVC leachates compared to 0% leachate control (AMP1 media). Growth curves (**a**–**d**) and growth rates (**e**, **f**) for each treatment are shown. Data points are mean values of triplicate biologically independent samples with error bars representing the standard deviation (error bars not visible where values were smaller than symbols). Lower case letters at the 72 h timepoints in **a**–**d** signify which treatments were found to be significantly different (*p* < 0.01) in population density at 72 h post exposure; source data and exact *p*-values for all timepoints are provided in Supplementary Data 1

Growth was impaired for both *Prochlorococcus* MIT9312 and NATL2A across the full range of HDPE and PVC leachate dilutions tested, as measured by flow cytometric counts of chlorophyll fluorescent cells (Fig. 1). MIT9312 responded more quickly to both HDPE and PVC leachate exposure than NATL2A. In MIT9312, significant (*p* < 0.01) population reductions were observed at 48 h for all tested HDPE and PVC leachate dilutions compared to the controls, whereas in NATL2A only the two most concentrated HDPE and PVC leachates were significantly different from controls at 48 h (*p*-values in Supplementary Data 1). After 72 h of exposure, the population density of both strains was reduced for all levels of HDPE and PVC leachate tested (Fig. 1a–d). For each plastic, higher concentrations of leachate resulted in greater reductions in population density, indicating that leachate exposure affected cell populations in a dose-dependent manner (Fig. 1a–d).

Population growth rates also declined in a dose-dependent manner across the three days of the experiment (Fig. 1e, f). Both strains of *Prochlorococcus* showed an overall negative growth rate across the experiment when exposed to HDPE leachate dilutions of 12.5 to 50%. PVC leachate exposure resulted in a markedly greater effect on MIT9312 growth compared to NATL2A. All tested PVC leachate dilutions resulted in negative growth rates for MIT9312, whereas NATL2A was able to maintain a positive growth rate for 0.25 to 1% PVC dilutions.

### Plastic leachates impair photosynthesis in *Prochlorococcus*

**Fig. 2:**
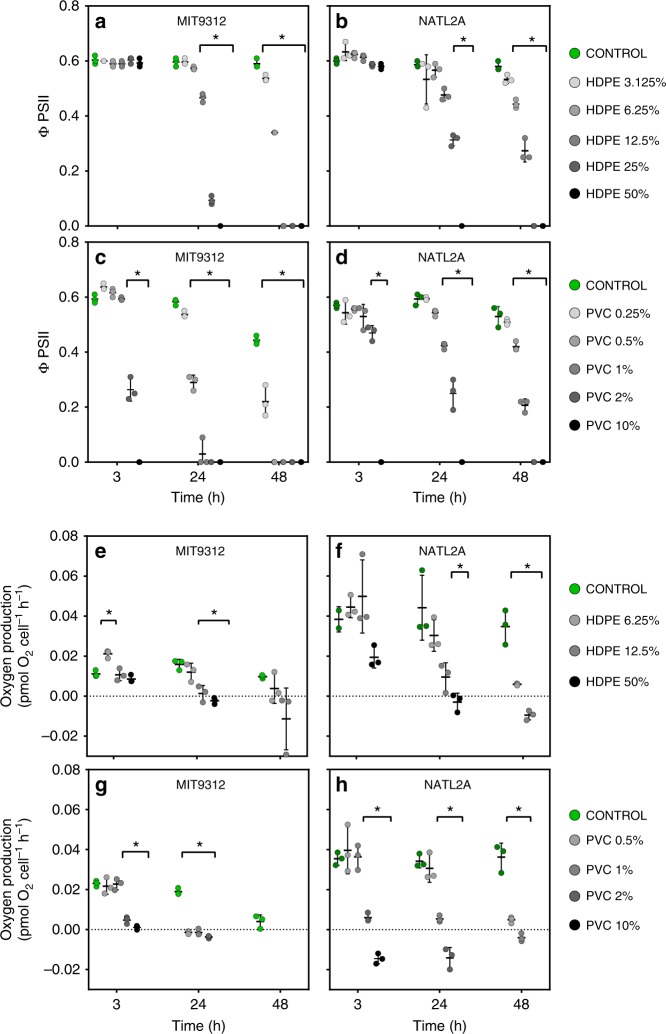
Photosynthetic capacity of *Prochlorococcus* MIT9312 and NATL2A in the presence of diluted HDPE and PVC leachates compared to 0% leachate control (AMP1 media). Measurements of effective quantum yield of PSII (Φ_PSII_) (**a**–**d**) and oxygen production rates (**e**–**h**) for *Prochlorococcus* MIT9312 and NATL2A control and leachate exposed populations after 3, 24 and 48 h of exposure. Data points are from triplicate independent biological samples (except for oxygen production rate measurements for NATL2A 3 h control in HDPE experiment where *n* = 2). In **e**–**h**, a subset of leachate dilutions were tested and once negative oxygen production rates were recorded (indicating respiration rates were likely higher than oxygen production rates) no more measurements were made. Asterisks are used to signify treatments for which measurements were significantly different (*p* < 0.01) to the control at the examined time point; source data and exact *p*-values for all timepoints are provided in Supplementary Data 2

In addition to effects on population growth, leachate exposure had a strong, dose-dependent effect on photochemical efficiency of photosystem II (PSII) in both *Prochlorococcus* MIT9312 and NATL2A (Fig. 2a–d). As with population growth, differences were observed with respect to dose sensitivity and timing of response of the two strains. The effective quantum yield of PSII (Φ_PSII_) was significantly (*p* < 0.01) reduced on HDPE leachate exposure in MIT9312 across all tested dilutions and all but the lowest concentration (3.125%) in NATL2A (Fig. 2a, b, Supplementary Table 2). Both strains showed significant reductions in Φ_PSII_ at 24 h for HDPE leachate dilutions of 12.5 to 50%. Strain-specific differences, however, were apparent at 48 h: Φ_PSII_ was affected in MIT9312 at all tested dilutions and had declined to undetectable levels for 12.5% HDPE leachate whereas Φ_PSII_ reductions were more moderate in NATL2A. Exposure to 10% PVC leachate (the highest tested concentration) resulted in a very rapid decline in effective quantum yield of PSII in both strains. Measurements of Φ_PSII_ 3 h post exposure showed that there was no detectable PSII activity in either strain in this treatment by this time. Intermediate and low PVC concentrations again resulted in more rapid and pronounced declines in Φ_PSII_ for MIT9312 than NATL2A and treatment with 0.25% PVC leachate did not significantly affect NATL2A Φ_PSII_ (Fig. 2c, d, *p*-values in Supplementary Table 2).

Clear declines in oxygen production rates were also observed following HDPE and PVC leachate exposure. For both strains exposed to HDPE leachates oxygen production rates started declining from 24 h (Fig. 2e, f, Supplementary Table 2). A slight increase in the oxygen production rate was observed in MIT9312 after 3 h exposure to 6.25% HDPE leachate, which may imply an initial effort to obtain energy when subjected to stress. Exposure to both 2 and 10% PVC leachate had a significant negative effect on oxygen production rates within 3 h in both strains (Fig. 2g, h, Supplementary Table 2). By 24 h, oxygen production had halted for MIT9312 cultures exposed to all tested PVC concentrations, whereas NATL2A continued to produce oxygen at similar levels to the control for 0.5% PVC until 48 h.

### Leachate exposure results in global transcriptional changes

Global transcriptomic analyses (RNA-Seq) were conducted to determine the early-stage transcriptional response to leachate exposure using dilutions that impaired population growth in both strains following 24–48 h exposure (50% HDPE; 2% PVC compared to no leachate controls). In MIT9312 exposure to leachates resulted in significant (*p* < 0.01) differential transcription of a large number of coding genes: 589 for PVC and 403 for HDPE, whereas a substantially lower proportion were impacted in NATL2A: 66 for PVC and 136 for HDPE (Supplementary Data 3 and 4). Highly responsive genes (log_2_ fold changes greater than ±1) were mapped onto Clusters of Orthologous Groups (COGs) and gene ontology (GO) categories to identify processes that were transcriptionally affected by the leachate exposure treatments (Supplementary Table 1).

**Fig. 3:**
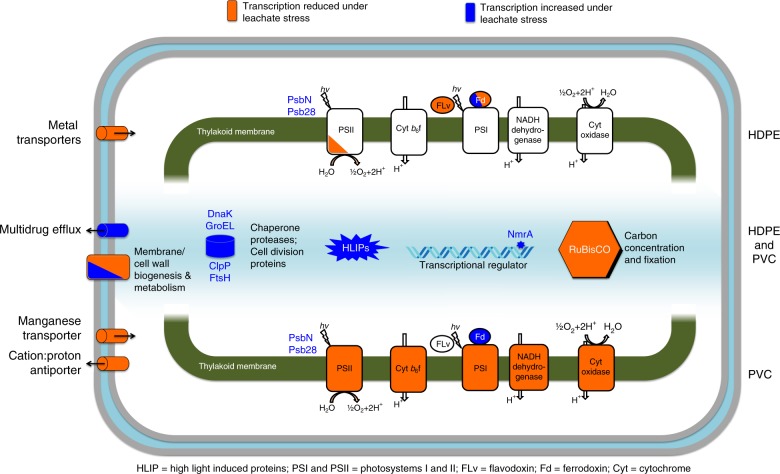
Schematic model of *Prochlorococcus* MIT9312 showing key genes and functions with significant responses to short-term HDPE and PVC plastic leachate exposure based on transcriptomic data. The sets of highly responsive genes in both MIT9312 and NATL2A organized by functional categories are listed in Supplementary Table 1 and complete lists of differentially transcribed genes for MIT9312 and NATL2A are found in Supplementary Data 3 and 4, respectively

Examination of highly transcriptionally responsive genes showed a number of processes were strongly influenced by leachate exposure in MIT9312 (Fig. 3, Supplementary Table 1). Exposure to both leachates led to increased transcription of common stress response genes, such as *groEL*, *dnaK*, *clpP* and *ftsH*. Both leachate treatments also resulted in strong positive transcriptional responses in a large number of *hli* (high-light inducible) family genes. In *Prochlorococcus* MED4, *hli* genes have been shown to be transcriptionally responsive to specific stress conditions, suggesting they have a role in oxidative and other stresses linked to photosynthesis^22^. An uncharacterized, *Prochlorococcus*-specific transcriptional regulator (PMT9312_RS06030) was also very strongly upregulated under exposure to both leachates and may have a role in coordinating the cellular response to plastic leachate-induced stress.

In MIT9312, genes associated with primary production were among the most highly affected following leachate exposure. Transcription was strongly reduced for a number of MIT9312 carbon fixation associated genes on exposure to both leachates, compared to the control (Fig. 3, Supplementary Table 1). Among the set showing the highest fold change was a cluster of five genes (PMT9312_RS02820–40) encoding carbon dioxide concentrating mechanism protein CcmK, both RUBISCO subunits and carboxysome shell proteins. Genes involved in photosynthesis and light-harvesting also showed clear transcriptional level changes under both leachate exposures, fitting with the observed photophysiological responses. PVC leachate exposure resulted in substantial reduction in transcription of almost all genes encoding photosystem I subunits (*psaA,B,C,D,E,F,J,K,L*), most photosystem II (*psbB,H,J,M,O,T,Z*) subunits, cytochrome b_6_-f complex subunits (petA,C,G), and photosynthetic pigment biosynthesis (Supplementary Data 3). By contrast, the MIT9312 transcriptional impact of HDPE leachate on photosynthesis-related genes was less pronounced, with transcription significantly reduced for *pcbD* encoding Chl *a/b* binding light-harvesting protein and photosystem II associated genes (*psbB,J,K,T,O* and PMT9312_RS06075 encoding PsbF-like protein), whereas two *psbA* genes showed a significant increase in transcription (Supplementary Data 3). In response to both PVC and HDPE plastic leachate treatments, genes implicated in photosystem repair (Psb28, reported to be involved in PSII repair mechanisms in cyanobacteria^23^ and PsbN, involved in repair from photoinhibition in higher plants^24^) were substantially upregulated.

The MIT9312 leachate transcriptional response also involved altered transcription of a number of transport and cell wall/membrane associated genes (Fig. 3, Supplementary Data 3). In both PVC and HDPE experiments, genes encoding putative multidrug efflux ABC transporter (PMT9312_RS04330) and tetracycline resistance major facilitator superfamily efflux pump showed significantly higher transcription in leachate exposed cultures relative to the control. In both instances, fold changes were higher for PVC leachate exposure than HDPE. In contrast to the efflux-associated genes, a number of other transporters showed differentially reduced transcription under leachate exposure conditions. HDPE leachate exposure resulted in reduced transcription of an iron ABC transporter substrate-binding protein PMT9312_RS06520, metal ABC transporter permease PMT9312_RS03095 and porin PMT9312_RS06535 relative to the no leachate control. PVC leachate exposure resulted in a relative reduction in transcription of a different set of genes which may also be associated with metal transport: a putative manganese ABC transporter PMT9312_RS03085-90, cation:proton antiporter PMT9312_RS08725 and anion transporter PMT9312_RS01090. A number of genes within the cell wall/membrane biogenesis functional category also showed strong reductions in transcription under leachate exposure conditions which may also affect movement of substances into and out of the cell.

In NATL2A, far fewer genes were significantly differentially transcribed. Of the genes that did show a strong transcriptional response to plastic leachate exposure, many are annotated as hypothetical proteins and do not have functions ascribed (Supplementary Data 4). Within the functionally annotated set of strongly differentially transcribed genes were a number of putative regulators that showed transcriptional increases in leachate exposed cultures (Supplementary Table 1). DNA-binding response regulator PMN2A_RS05195 increased under HDPE exposure, whereas GntR family transcriptional regulator PMN2A_RS09350 increased under PVC exposure, indicating that regulation of the leachate exposure response may be mediated by different genes in each strain (Supplementary Table 1). Other significant transcriptional changes observed in NATL2A include reduced transcription of genes involved with nitrogen metabolism under HDPE leachate exposure with substantial decreases observed for genes encoding a ferredoxin-nitrite reductase, nitrite transporter, urea ABC transporter substrate-binding protein (PMN2A_RS09785, RS09790, RS08475) as well as the global nitrogen regulator NtcA (RS01460). PVC leachate exposure impacted transcription of genes associate with cell wall/membrane biogenesis and transport, such as sugar transferase (PMN2A_RS07615) and a porin (PMN2A_RS02250).

### Leachates are a mixture of organic and inorganic substances

Exploratory analyses were performed on the HDPE and PVC leachates to investigate their composition and attempt to determine possible contributors to the observed growth and transcriptomic impacts. Untargeted Liquid Chromatography–Mass Spectrometry (LC–MS) analysis of the organics in the leachates indicated that the plastic leachates contained a complex mixture of organic components, whilst not identifying specific compounds. The HDPE and PVC leachates contained 5877 and 10658 components, respectively, that were absent or observed at substantially lower levels in the AMP1 media. Of these, 5799 were observed in the leachates from both plastics, with PVC leaching a number of additional components. Many components also appeared to be more highly abundant in the PVC leachate compared to either the HDPE leachate or the basal AMP1 media (Supplementary Fig. 1).

Although the broad chemical screening applied did not allow identification of individual chemical species, library searches of the mzCloud mass spectral database and Compound Discoverer Extractables and Leachables mass list were performed. The most highly enriched leachate components had no matches in either database. Database matches (tentative identifications) were obtained for a relatively small number of strongly enriched (log_2_ fold > 2) components in the PVC and HDPE leachate (168 and 53, respectively, Supplementary Data 5).

Leachate elemental composition analyses conducted using Inductively Coupled Plasma–Optical Emission Spectrometry and Mass Spectrometry (ICP–OES and ICP-MS) identified elements enriched in leachates relative to basal medium. Zinc (Zn) was observed at higher concentrations in HDPE leachate than AMP1 basal medium (~31X). HDPE leachate also contained manganese and nickel at concentrations above the method detection limit (MDL), whereas levels in the control were below this level, indicating that these are also enriched to some degree in these leachates.

In the PVC leachate Zn was highly enriched (~564X) relative to basal media, and strontium levels were also enriched (~7X higher than basal media) (Supplementary Table 2). Copper was also measured at levels above the method detection limit (MDL) in the PVC leachate sample but was below this limit in the control suggesting some degree of enrichment.

## Discussion

Here we report that exposure to leachate from two common plastic items, HDPE bags and PVC matting is linked to significant impairment of *Prochlorococcus* in vitro growth and photosynthetic capacity. From this study, it is clear that different plastics vary considerably in their potential toxicity, consistent with what has been observed in previous eukaryote focused studies^5^. Exposure to PVC leachates affected *Prochlorococcus* to a greater degree than HDPE leachates, reflected in considerably less PVC leachate required to negatively affect *Prochlorococcus* growth and photophysiological responses. This was consistent with the exploratory chemical analysis of these leachates, which indicated that the PVC matting leached a larger variety and abundance of organic substances, as well as notably higher levels of Zn than the tested HDPE bags. PVC manufacture is reported to use a relatively larger proportion of various additives, including plasticisers (for example, phthalates), heat stabilisers (based on Pb, Sn, Ba, Cd and Zn compounds) and biocides than other plastic types^6^.

Although this study did not identify the organic substances contained within the plastic leachates, marine *Prochlorococcus* have previously been found to be highly sensitive to other organic pollutants^21,25^. Polar and non-polar organic pollutants, particularly when present as mixtures were found to exert toxic effects on oceanic phytoplankton, with picocyanobacteria *Synechococcus* and *Prochlorococcus* found to be particularly strongly affected^20,25^. Echeveste and colleagues^21^ investigated the toxicity of model Polycyclic Aromatic Hydrocarbons (PAHs) pyrene and phenanthrene to both laboratory cultures and natural communities of phytoplankton. Of the tested culture isolates, *Prochlorococcus* was found to be particularly sensitive to these PAHs, presumably due to their smaller cell size and therefore relatively higher surface area to volume ratio. More recently, Echeveste and colleagues^25^ have shown that complex mixtures of persistent organic pollutants (POPs) concentrated from seawater have greater toxicity than single or simple POP mixtures and that ocean populations of *Prochlorococcus* and *Synechococcus* are particularly strongly affected. It has also been shown that exposure to relatively low levels of organic pollutants influences photosynthesis in *Prochlorococcus*, with decreased transcription of genes encoding RuBisCO large subunit and PSII D1 protein reported in marine populations exposed to non-polar compounds^17^. Although it is unclear if there is any overlap between these organic pollutants tested previously and what might be present in the plastic leachates used in our treatments, these findings do suggest that *Prochlorococcus* are sensitive to at least some organic pollutants. Thus, some part of the response observed for *Prochlorococcus* may be associated with organic substances released by plastic items into the environment.

Elemental characterisation indicated that some metals are also enriched in plastic leachate. Of the examined elements, zinc showed the highest degree of enrichment in both leachates and were particularly high in PVC leachate. Zinc is a known component of many plastic additives, including slip agents, colorants, fillers and heat stabilisers, with the latter used mainly in PVC products^6^. Although to our knowledge there are no published studies looking directly at Zn toxicity in *Prochlorococcus*, such studies have been conducted in *Synechococcus* strains. Exposure of natural populations of closely related marine cyanobacteria *Synechococcus* to a range of Zn concentrations found the highest tested concentration, 713 μg L^−1^, resulted in low-to-moderate growth inhibition^26^. By comparison, the elemental analysis in this study indicates the Zn concentration was ~628 μg L^−1^ for the 10% PVC leachate and ~173 μg L^−1^ for 50% HDPE leachate, the least diluted (highest concentration) treatments tested for each. Freshwater *Synechococcus* sp. IU625 was found to tolerate exposure to 10 mg L^−1^ ZnCl_2_, whereas concentrations of 25 mg L^−1^ reduced growth to ~50% after 7 days exposure^27^. Furthermore, transcriptomic analyses of *Synechococcus* sp. IU625 indicated that the relatively high tolerance exhibited by this strain was largely mediated by membrane modifications and probable Zn efflux back into the environment, as porins, permeases and substrate-binding proteins showed increased transcription following seven days exposure to 10 mg L^−1^ ZnCl_2_. It is important to note, however, that sensitivity to metals varies considerably at the strain level in cyanobacteria and marine cyanobacteria are particularly sensitive^28^, so additional work to look directly at *Prochlorococcus* Zn sensitivity would be required to determine what component of the leachate stress response is linked to the presence of Zn.

The two *Prochlorococcus* strains in this study showed different growth and photophysiology responses to both leachates, but particularly for PVC leachate. HLII ecotype *Prochlorococcus* MIT9312 responded more rapidly to all leachates. The transcriptomic responses of MIT9312 to short-term leachate exposure also involved considerably more genes and different global regulatory genes than LLI ecotype NATL2A. This implies that distinct regulatory networks are co-opted in each strain in response to leachate exposure, which may have contributed to the observed differences in physiological response. Similarly, distinct responses have been observed in previous studies comparing the transcriptomic responses of different *Prochlorococcus* strains to environmental perturbations. For example, only four of more than 1000 orthologs were differentially expressed in *Prochloroccoccus* MED4 (HLI ecotype) and MIT9313 (LLIV ecotype) in response to low iron availability^29^. Given that differences were observed in the degree of sensitivity of *Prochlorococcus* strains to plastic leachates and that each mounted a distinct transcriptomic response, we suggest that leachate exposure will affect some photosynthetic marine organisms to a greater extent than others, and thus potentially influence microbial community composition.

It is not possible to equate our laboratory experiments with a specific concentration of plastic in the ocean, but it is clear that marine organisms, including *Prochlorococcus*, will increasingly encounter plastic particles in their environment. There are estimated to be ~1.8 trillion plastic pieces within the 1.6 million km^2^ ‘Great Pacific Garbage Patch’ region of the North Pacific Subtropical Gyre (NPSG)^3^, where *Prochlorococcus* is the most abundant phototroph, reaching 10^8^ cells L^−1^ in the surface mixed layer^14,15^. Current trends indicate mismanaged plastic waste will increase up to 10-fold over the next ten years^4^, suggesting that the marine plastic burden and its ecological impact will continue to escalate. There is recent evidence that plastic leachates can be a source of dissolved organic carbon and promote growth of some heterotrophic marine microbes in vitro^13^. However, our results indicate that some concentrations of leachates from common plastic items also have the capacity to impair functioning of photosynthesis in *Prochlorococcus* and may adversely affect other important marine bacteria. This in vitro study provides an important preliminary step in understanding how ecologically significant photosynthetic bacteria respond to plastic leachates. By considering the effects of marine plastic pollution on key marine microbes, we have the potential to develop improved risk assessment frameworks addressing this burgeoning global issue.

## Methods

### Cell culturing conditions

*Prochlorococcus* MIT9312 from HLII ecotype and NATL2A from LLI ecotype were cultured in acid washed, sterile borosilicate glassware using the artificial seawater medium AMP1, which consists of a base of Turks Island Salt Mix to which macronutrients, trace metals and HEPES buffer are added^30^. Three independent biological replicates were used for all experiments. Before experiments commenced, cells were acclimated at 22 °C in a shaking incubator at 100 rpm with continuous illumination of ~20 µmol photons m^−2^ s^−1^ (NATL2A) and ~40 µmol photons m^−2^ s^−1^ (MIT9312) (Infors HT Multitron). Cell growth was regularly monitored via measurement of in vivo chlorophyll a fluorescence as a proxy for biomass (BMG Pherastar) and via optical density at 750 nm to ensure balanced exponential growth under light and temperature conditions prior to starting each experiment. Acclimated, exponential phase grown cultures were used as the inoculum in all experiments.

### Leachate preparation

Grey plastic grocery bags (HDPE) and textured black and yellow/green plastic matting (PVC) were sourced from local shops for use in this study. Leachate generation procedures were based on methods used in previous leachate toxicity testing studies^9,10^. All plastic items were unused, free of visible dust and were used without washing to test the first leaching. Plastic materials were cut into ~1–2 cm^2^ pieces (Supplementary Fig. 2) using clean, stainless steel scissors and 5 g added to 100 mL of Turks Island Salt Mix^31^ in acid washed, sterile borosilicate flasks, then allowed to leach for five days under the same growth irradiance, temperature and mixing conditions described for cell culturing. In order to obtain sterile, particle free leachate in Turk’s Island Salt Mix following the leaching period, the contents of each flask were filtered through a sterile 0.2 µm filter unit (Corning), that was rinsed first with sterile, ultrapure H_2_O (Millipore). To each volume of filtered leachate, the standard AMP1 macronutrients, trace metals and HEPES buffer were then aseptically added to make HDPE and PVC leachate stocks. For each plastic testing was performed using five leachate dilutions prepared in AMP1. For HDPE vol/vol dilutions of 50, 25, 12.5, 6.25 and 3.125% in AMP1 were used, representing ~25–1.6 mg mL^−1^ (or ~1.45–0.007 pieces per mL of media). For PVC vol/vol dilutions of 10, 2, 1, 0.5 and 0.25% were used, representing ~5–0.125 mg mL^−1^ (or ~0.02–0.0004 pieces per mL of media). The regular AMP1 media served as the 0% leachate control. Subsamples of HDPE and PVC leachate-containing and 0% leachate control AMP1 media were collected in acid washed glass vials for Liquid Chromatography–Mass Spectrometry (LC–MS) and similarly for Inductively Coupled Plasma–Mass Spectrometry (ICP–MS) and Inductively Coupled Plasma–Optical Emission Spectrometry (ICP–OES).

### Flow cytometry

The concentration of *Prochlorococcus* populations in experimental flasks was quantified using a CytoFLEX S flow cytometer and CytExpert software (Beckman Coulter). *Prochlorococcus* cells were identified by chlorophyll fluorescence using blue laser (488 nm) excitation and violet side angle light scattering properties using violet laser (405 nm) excitation. Samples were gated so that only cells with chlorophyll fluorescence intensities indicative of healthy cells were counted (Supplementary Fig. 3). At each time point throughout the experiment 50 µL of cells were collected from each flask into 450 µL sterile, filtered AMP1 media and immediately fixed with paraformaldehyde (final concentration of 1%), incubated in the dark at 4 °C for 1 h, then frozen in −80 °C until later analysis.

### Functional chlorophyll *a* fluorescence measurements

Photochemical efficiency was determined using a Phytoplankton Pulse Amplitude Modified (Phyto-PAM) Fluorometer (Walz GmbH, Effeltrich, Germany), applying the saturation pulse technique^32^. Strain-specific growth light conditions were used to measure light adapted fluorescence yield (F) and a saturation pulse applied to determine maximum light adapted fluorescence yield (*F*_m_′), to calculate the effective quantum yield of photosystem II, Φ_PSII_ (Φ_PSII_ = [*F*_m_′ − *F*]/*F*_m_′)^33^. Three technical replicates were run for each individual biological replicate, and an average of these for each biological replicate was used to calculate the average and standard deviation presented for each time point.

### Oxygen production rate measurements

Oxygen production rates were measured using a fibre optic oxygen sensor (Pyroscience^R^ Firesting O_2,_ Germany) to detect oxygen levels at integrated optical oxygen sensor spots (REDFLASH® indicator) within the respiration vial. A 2-point calibration was performed following the integrated calibration procedure of the Pyroscience software ‘Profix’, measuring 100% oxygen (filtered AMP1 bubbled with ambient air for ~30 min) and 0% oxygen (sodium dithionite added to filtered AMP1) prior to experimental measurements. Three respiration vials were overfilled with experimental subsamples (avoiding any enclosed bubbles when closing the vial) and submerged in a water jacket adjusted to the culture rearing temperature of 22 °C. Oxygen production was recorded using ‘Profix’ during light incubations (100 µmol photons m^−2^ s^−1^ using LED light strips; Ecolamp (Havit Lighting), 46W, cool white), collecting readings for 5 to 10 min after steady rates were observed. Rates were normalised using flow cytometric cell counts for the same culture.

### RNA isolation, sequencing and analysis

Total RNA was extracted from triplicate 50 mL cultures grown in control (AMP1) and leachate-containing media (50% dilution HDPE leachate, 2% dilution PVC leachate) after short-term exposure (90 min for PVC, 120 min for HDPE) using the Qiagen MiRNeasy kit according to the manufacturer’s instructions. RNA samples were depleted of ribosomal RNA using the Ribozero Bacteria kit (Illumina). TruSeq Stranded RNA-Seq libraries were prepared and sequenced using NextSeq500 75 bp SR (Illumina) at the Ramaciotti Centre for Genomics (UNSW, Sydney, Australia). The quality of reads was assessed with FastQC, and EDGE-pro (Estimated Degree of Gene Expression in PROkaryotes) v1.3.1^34^ was used to align reads to genome sequences (NCBI references sequences NC_007577.1 and NC_007335.2 for MIT9312 and NATL2A, respectively). The Subread featurecounts script (v1 in 1.5.2) was used to summarize reads to genomic features^35^ and the R package DESeq2^36^ was used to identify differential expression between control and leachate exposed cultures, applying an adjusted (Benjamin–Hochberg) cutoff of *p* < 0.01 to compile a list of differentially transcribed genes. Raw and analyzed transcriptomic data were submitted to the NCBI GEO database, accession number GSE118155.

### Leachate composition analyses

Leachate samples for elemental composition analyses were acidified with 3.5% nitric acid prior to ICP–OES, and ICP-MS analysis performed on a NexION 300D (Perkin Elmer) instrument at the Mark Wainwright Analytical Centre, UNSW, Sydney Australia. For each of the independent samples, three technical replicates were run, and internal standards were used in each run (Rh, Ir and Te from Choice Analytical, Australia).

Exploratory, untargeted analyses of the organic composition of leachates were performed with high resolution LC–MS/MS using a U3000 UHPLC system interfaced to a Q-Exactive Plus mass spectrometer (ThermoFisher Scientific) at the Mark Wainwright Analytical Centre, UNSW, Sydney Australia. Ten technical replicates were run for each sample (AMP1 only, as a background media ‘blank’, HDPE leachate in AMP1 and PVC leachate in AMP1) to permit statistical analysis of the data. For each replicate 100 µL (in randomized order) was injected onto a Waters BEH C18 UPLC column (100 × 2.1 mm) and chromatographed over 30 min using a linear gradient of 0.1% formic acid in water against acetonitrile at 400 µL min^−1^. Column eluate was directed into the heated electrospray ionisation source of the mass spectrometer. Mass spectra were acquired over the range 100–1100 in positive ion mode approximately every 1 s at a resolution of 60,000. Automated MS/MS analysis was performed on the most intense ions observed in the spectra. Data were interrogated using Compound Discoverer version 2.1 (ThermoFisher Scientific) software. This involved grouping technical replicates for each sample, alignment of chromatograms and correction for retention time drift across analysis, then performing peak detection using the default settings (intensity tolerance of 30 and a signal to noise threshold of 3) (resulting peak data is provided in Supplementary Data 6). To screen for components detected with high confidence in the HDPE and PVC leachates, data was filtered to include only detected peaks that fit all of the following criteria. The coefficient of variation (CV) percentage was ≤ 40 (removing species that showed high variability between technical replicates in all samples). The peak area was significantly different (*p*-value ≤ 0.001) and larger in area (peak area ratio > 5) in the leachate sample compared to the AMP1 blank. Components arising from the media solution or from sample processing were therefore removed from consideration. This set of leachate components were then used in database searches of both the mzcloud online MS/MS library and the Extractables and Leachables mass list database within Compound Discoverer.

### Statistics and reproducibility

For primary growth, photophysiology and transcriptomic experiments three independent biological replicate cultures were acclimated and transfers from each were used as replicates in each experiment. Assays were highly reproducible for all measurements collected and preliminary experiments indicated that this level of replication provided minimal variability between replicates allowing robust statistical analysis. All growth and photophysiology data were analysed with one-way ANOVA followed by Tukey’s multiple comparison test with 95% confidence intervals using GraphPad Prism 7 for Windows (GraphPad Software, La Jolla California USA, www.graphpad.com).

### Reporting summary

Further information on research design is available in the Nature Research Reporting Summary linked to this article.

## Supplementary information

Supplementary Information

Description of Additional Supplementary Files

Reporting Summary

Supplementary Data 1

Supplementary Data 2

Supplementary Data 3

Supplementary Data 4

Supplementary Data 5

Supplementary Data 6

## Data Availability

All data are available in the main text or the Supplementary Information. Sequence data can be downloaded from NCBI GEO database, accession number GSE118155.
