# Supplementary material for: Plastic leachates impair growth and oxygen production in Prochlorococcus, the ocean’s most abundant photosynthetic bacteria
Source: Commun Biol. 2019 May 14;2:184. doi: 10.1038/s42003-019-0410-x (PMC6517427; doi:10.1038/s42003-019-0410-x)
Supplement: Supplementary file 1 — Supplementary Information [file 42003_2019_410_MOESM1_ESM.pdf]

Supplementary Figures

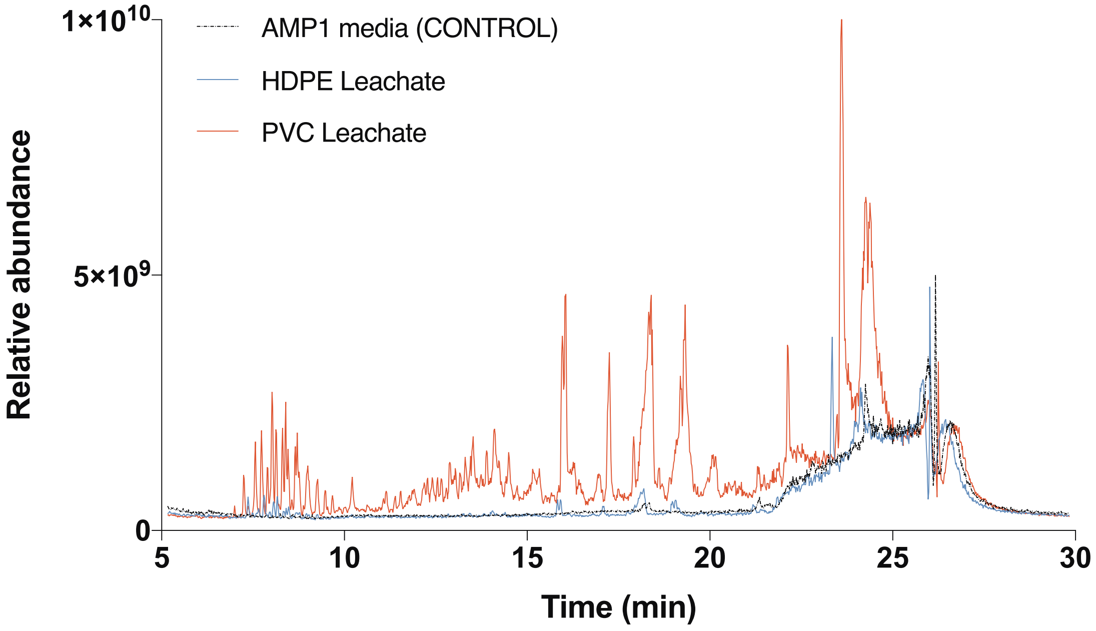

Supplementary Figure 1: Overlaid representative Liquid Chromatography – Mass Spectrometry total ion chromatograms for AMP1 media (control), HDPE and PVC leachates. The chromatograms illustrate the differences in signal, with a number of components highly abundant in the PVC leachate relative to both the HDPE leachate and the AMP1 media control (the rise in baseline from 22-27 minutes in all samples is primarily due to the change in mobile phase gradient).

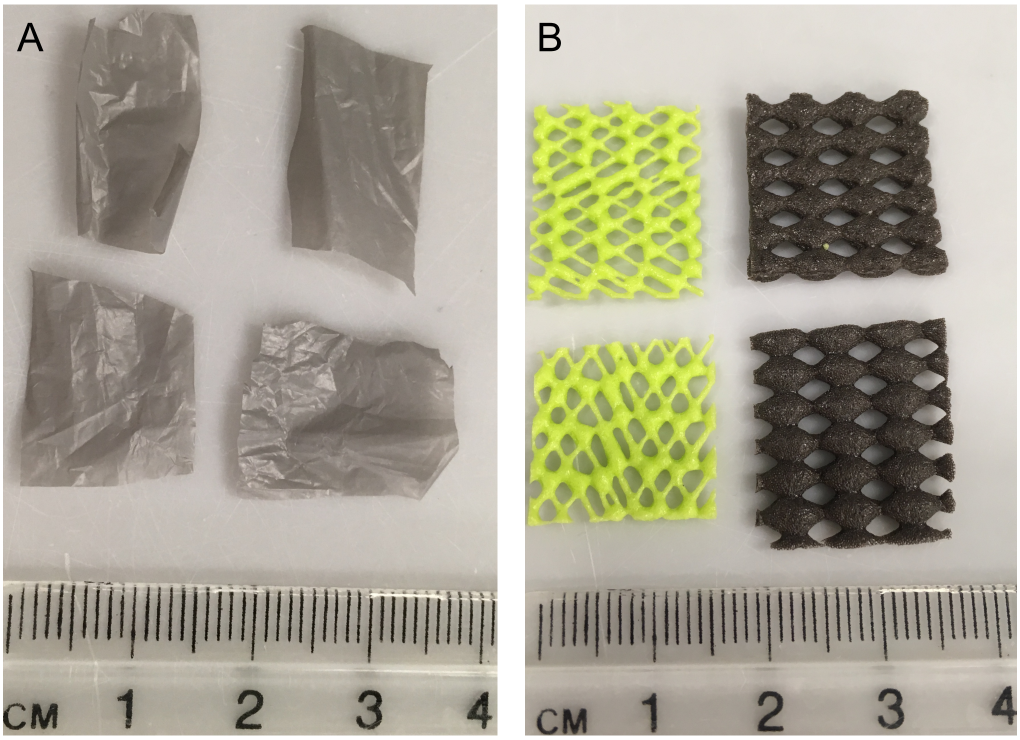

Supplementary Figure 2: Examples of plastic item pieces used to generate leachates. A. HDPE grey single-use shopping bag pieces and B. PVC matting (mix of yellow/green and black pieces).

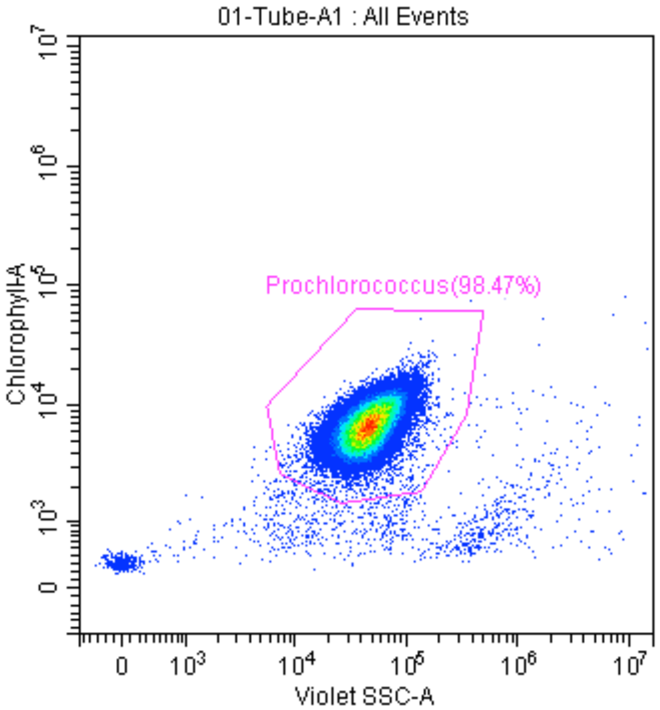

Supplementary Figure 3: Flow cytometry gating strategy. Gating strategy used to determine flow cytometry counts of *Prochlorococcus* population for determination of Chlorophyll *a* autofluorescent cell density in control and plastic leachate (HDPE and PVC) exposed culture. No marker or fluorochrome was used in assays. Cells inside gate "Prochlorococcus" represent Chlorophyll *a* autofluorescent cells counted as healthy cells for the assay. The percentage of total cells within the gated population is shown on the figure. This gating strategy was used for all cell counts, including cells that were dying, as defined by lower Chlorophyll *a* autofluorescence.

Supplementary Tables

Supplementary Table 1: Selected *Prochlorococcus* functional categories strongly differentially expressed following short-term leachate exposure. The functional categories presented were selected based on the presence of strongly (log2 fold change > +/-1) differentially transcribed genes. Within these categories additional differentially transcribed genes with log2FC > +/- 0.8 are included for MIT9312 and +/- 0.5 for NATL2A are included in this table. The full set of signficantly differentially transcribed genes and log 2 fold change data are given in Supplementary Dataset 3 (MIT9312) and Supplementary Dataset 4 (NATL2A).

		Prochlorococcus MIT9312		*Prochlorococcus* NATL2A	
	**Functional categories***	**HDPE leachate**	**PVC leachate**	**HDPE leachate**	**PVC leachate**
**Majority showing increased trascript abundance in leachate exposed cells**	**Post-translational modification, protein turnover, chaperone functions**	PMT9312_RS07890 molecular chaperone GroEL,<br>PMT9312_RS09260 molecular chaperone DnaK,<br>PMT9312_RS07285 ATP-dependent Clp protease proteolytic subunit,<br>PMT9312_RS07290 ATP-dependent Clp protease proteolytic subunit,<br>PMT9312_RS07025 cell division protein FtsH	PMT9312_RS01820 peroxiredoxin,<br>PMT9312_RS01150 cell division protein FtsH,<br>PMT9312_RS03860 cell division protein FtsH,<br>PMT9312_RS07285 ATP-dependent Clp protease proteolytic subunit,<br>PMT9312_RS07290 ATP-dependent Clp protease proteolytic subunit,<br>PMT9312_RS07025 cell division protein FtsH,<br>PMT9312_RS08155 serine protease,<br>PMT9312_RS00570 glutathione S-transferase,<br>PMT9312_RS00090 SirA family protein,<br>PMT9312_RS08075 molecular chaperone DnaJ,<br>PMT9312_RS05220 glutathione peroxidase,<br>PMT9312_RS07890 molecular chaperone GroEL,<br>PMT9312_RS08005 cytochrome c biogenesis protein CcsB,<br>PMT9312_RS09260 molecular chaperone DnaK,<br>PMT9312_RS08000 cytochrome C biogenesis protein CcdA,<br>PMT9312_RS05660 ATP-dependent Clp protease ATP-binding subunit ClpC,<br>#PMT9312_RS03965 cytochrome c biogenesis protein CcsA<br>#PMT9312_RS09020 ATP-dependent Clp protease ATP-binding subunit ClpX		
	**Putative regulatory proteins**	PMT9312_RS06030 NmrA family transcriptional regulator	PMT9312_RS06030 NmrA family transcriptional regulator,<br>PMT9312_RS09225 RNA polymerase sigma factor RpoD,<br>PMT9312_RS00790 helix-turn-helix transcriptional regulator	PMN2A_RS05195 DNA-binding response regulator,<br>PMN2A_RS04310 SOS mutagenesis protein UmuD,<br>#PMN2A_RS01460 global nitrogen regulator NtcA	PMN2A_RS09350 GntR family transcriptional regulator,<br>PMN2A_RS01080 DNA-binding response regulator,<br>PMN2A_RS04925 NmrA family transcriptional regulator
	**High light inducible proteins**	PMT9312_RS03520, PMT9312_RS03530, PMT9312_RS03535, PMT9312_RS05815, PMT9312_RS06055, PMT9312_RS06345, PMT9312_RS06350, PMT9312_RS06355, PMT9312_RS07650, PMT9312_RS07655, PMT9312_RS07660, PMT9312_RS07800, PMT9312_RS07805, PMT9312_RS07795	PMT9312_RS03520, PMT9312_RS03530, PMT9312_RS03535, PMT9312_RS03605, PMT9312_RS05815, PMT9312_RS05895, PMT9312_RS06055, PMT9312_RS06345, PMT9312_RS06350, PMT9312_RS06355, PMT9312_RS06405, PMT9312_RS07305, PMT9312_RS07650, PMT9312_RS07655, PMT9312_RS07660, PMT9312_RS07795, PMT9312_RS07800, PMT9312_RS07805	PMN2A_RS05975	PMN2A_RS05975 PMN2A_RS07920 PMN2A_RS05085 PMN2A_RS07910
	**Defense mechanisms**	PMT9312_RS04330 ABC transporter ATP-binding protein (multidrug efflux - transportDB),<br>PMT9312_RS05055 HNH endonuclease	PMT9312_RS02880 ABC transporter ATP-binding protein,<br>PMT9312_RS04330 ABC transporter ATP-binding protein (multidrug efflux -transportDB)		PMN2A_RS00440 ABC transporter ATP-binding protein

		Prochlorococcus MIT9312		*Prochlorococcus* NATL2A	
	**Functional categories***	**HDPE leachate**	**PVC leachate**	**HDPE leachate**	**PVC leachate**
**Majority showing decreased transcript abundance in leachate exposed cells**	**Carbon dioxide fixation and concentration**	PMT9312_RS02830 ribulose bisphosphate carboxylase small subunit<br>PMT9312_RS02840 carboxysome shell protein<br>PMT9312_RS02825 ribulose bisphosphate carboxylase large chain<br>PMT9312_RS02835 carboxysome shell protein<br>PMT9312_RS02820 carbon dioxide-concentrating mechanism protein CcmK	PMT9312_RS02830 ribulose bisphosphate carboxylase small subunit<br>PMT9312_RS02840 carboxysome shell protein<br>PMT9312_RS02825 ribulose bisphosphate carboxylase large chain<br>PMT9312_RS02835 carboxysome shell protein<br>PMT9312_RS02820 carbon dioxide-concentrating mechanism protein CcmK		
	**Photosynthesis and chlorophyll biosynthesis**	PMT9312_RS06570 chlorophyll a/b binding light-harvesting protein,<br>PMT9312_RS06075 PsbF-like protein,<br>PMT9312_RS01385 photosystem II reaction center protein K (psbK),<br>PMT9312_RS07950 ferredoxin,<br>#PMT9312_RS07490 ferredoxin,<br>#PMT9312_RS04470 photosystem II reaction center protein Psb28,<br>#PMT9312_RS01280 photosystem II reaction center protein PsbN	PMT9312_RS02410 photosystem I PsaF protein (subunit III) (psaF)<br>PMT9312_RS02405 photosystem I reaction center subunit IX (psaJ)<br>PMT9312_RS08610 photosystem I protein PsaD (psaD)<br>PMT9312_RS08770 photosystem I iron-sulfur center protein (psaC)<br>PMT9312_RS04570 photosystem I reaction center subunit PsaK (psaK)<br>PMT9312_RS02370 apocytochrome f<br>PMT9312_RS08315 photosystem I P700 chlorophyll a apoprotein A2 (psaB)<br>PMT9312_RS08300 photosystem I reaction center protein subunit XI (psaL)<br>PMT9312_RS08320 photosystem I P700 chlorophyll a apoprotein A1 (psaA)<br>PMT9312_RS01725 photosystem I reaction center subunit IV (psaE)<br>PMT9312_RS01160 photosystem II manganese-stabilizing protein (psbO)<br>PMT9312_RS01620 photosystem II reaction center protein T (psbT)<br>PMT9312_RS01625 photosystem II chlorophyll-binding protein CP47 (psbB)<br>PMT9312_RS01635 photosystem II reaction center protein M (psbM)<br>PMT9312_RS01275 photosystem II reaction center protein H (psbH)<br>PMT9312_RS03215 chlorophyll a/b binding light-harvesting protein<br>PMT9312_RS02795 ferredoxin:protochlorophyllide reductase (ATP-dependent) subunit B,<br>PMT9312_RS02800 ferredoxin:protochlorophyllide reductase (ATP-dependent) subunit N,<br>PMT9312_RS02790 ferredoxin:protochlorophyllide reductase (ATP-dependent) iron-sulfur ATP-binding protein,<br>PMT9312_RS03940 geranylgeranyl reductase<br>#PMT9312_RS04470 photosystem II reaction center protein Psb28,<br>#PMT9312_RS01280 photosystem II reaction center protein PsbN		

		Prochlorococcus MIT9312		*Prochlorococcus* NATL2A	
	**Functional categories***	**HDPE leachate**	**PVC leachate**	**HDPE leachate**	**PVC leachate**
	**Amino acid metabolism and transport**	PMT9312_RS04155 ABC transporter ATP-binding protein (afuC),<br>PMT9312_RS01080 hypothetical protein	PMT9312_RS08605 anthranilate synthase component I (trpE),<br>PMT9312_RS09235 phospho-2-dehydro-3-deoxyheptonate aldolase,<br>PMT9312_RS01295 3-isopropylmalate dehydratase small subunit (leuD),<br>PMT9312_RS04120 1-(5-phosphoribosyl)-5-((5-phosphoribosylamino)methylideneamino)imidazole-4- carboxamide isomerase (hisA),<br>PMT9312_RS04645 N-acetyl-gamma-glutamyl-phosphate reductase,<br>PMT9312_RS04345 carbamoyl phosphate synthase small subunit,<br>PMT9312_RS04340 anthranilate phosphoribosyltransferase,<br>PMT9312_RS03460 pyridoxal phosphate-dependent aminotransferase,<br>PMT9312_RS00420 phosphoadenosine phosphosulfate reductase,<br>PMT9312_RS04505 alanine--glyoxylate aminotransferase family protein,<br>PMT9312_RS01080 hypothetical protein,<br>PMT9312_RS09265 shikimate dehydrogenase,<br>#PMT9312_RS02545 carboxypeptidase M32<br>#PMT9312_RS04250 urea ABC transporter substrate-binding protein (urtA)<br>#PMT9312_RS03045 peptidase M3 (prlC)	PMN2A_RS08475 urea ABC transporter substrate-binding protein, PMN2A_RS08470 branched-chain amino acid ABC transporter permease,<br>PMN2A_RS02625 acetylglutamate kinase,	PMN2A_RS02625 acetylglutamate kinase
	**Inorganic ion transport and metabolism**	PMT9312_RS06520 iron ABC transporter substrate-binding protein,<br>#PMT9312_RS04160 ferritin	PMT9312_RS02790 ferredoxin:protochlorophyllide reductase (ATP-dependent) iron-sulfur ATP-binding protein (chlL),<br>PMT9312_RS00835 rhodanese,<br>PMT9312_RS03090 manganese transporter (mntA),<br>PMT9312_RS08725 cation:proton antiporter,<br>PMT9312_RS03085 metal ABC transporter substrate-binding protein (mntC),<br>PMT9312_RS01090 sodium-independent anion transporter,<br>PMT9312_RS04185 2Fe-2S)-binding protein,<br>#PMT9312_RS02640 hypothetical protein<br>#PMT9312_RS09085 hypothetical protein<br>#PMT9312_RS03710 phosphate ABC transporter substrate-binding protein PstS	PMN2A_RS09785 ferredoxin--nitrite reductase,<br>PMN2A_RS09790 nitrite transporter	
	**Cell wall/membrane biogenesis**	PMT9312_RS02990 NAD(P)-dependent oxidoreductase,<br>PMT9312_RS07400 lipid-A-disaccharide synthase,<br>#PMT9312_RS03925 D-Ala-D-Ala dipeptidase	PMT9312_RS09300 glycosyl transferase,<br>PMT9312_RS06780 nucleotide sugar dehydrogenase,<br>PMT9312_RS06715 nucleotide sugar dehydrogenase,<br>PMT9312_RS06800 GDP-mannose 4,6-dehydratase,<br>PMT9312_RS06805 UDP-glucose 4-epimerase (galE),<br>PMT9312_RS02990 NAD(P)-dependent oxidoreductase,<br>PMT9312_RS09030 glycosyl transferase family 2	PMN2A_RS07615 sugar transferase	PMN2A_RS07615 sugar transferase,<br>PMN2A_RS09415 GDP-mannose 4,6-dehydratase
**Mix of increased and decreased transcript abundance in leachate exposed cells**	**Lipid transport and metabolism**	PMT9312_RS07635 fatty acid desaturase,<br>PMT9312_RS07545 short-chain dehydrogenase/reductase,<br>PMT9312_RS05760 1-acyl-sn-glycerol-3-phosphate acyltransferase	PMT9312_RS00310 acetyl-CoA carboxylase biotin carboxylase subunit,<br>PMT9312_RS07410 beta-hydroxyacyl-ACP dehydratase,<br>PMT9312_RS07635 fatty acid desaturase,<br>PMT9312_RS03175 acetyl-coenzyme A synthetase,<br>PMT9312_RS05760 1-acyl-sn-glycerol-3-phosphate acyltransferase,<br>PMT9312_RS02120 short-chain dehydrogenase,<br>PMT9312_RS07545 short-chain dehydrogenase/reductase,<br>PMT9312_RS09095 acyl-CoA desaturase	PMN2A_RS03225 acetyl-coenzyme A synthetase	PMN2A_RS06370 1-acyl-sn-glycerol-3-phosphate acyltransferase

*Categories assigned based on COG, GO or NCBI annotatation.

Genes for which transcriptional change was in the opposite direction to the main trend observed for this category. Colouring is consistent with Fig. 3 (blue and orange indicates genes for which transcription was signficantly higher and lower under leachate exposure, respectively).

Supplementary Table 2: Concentrations of selected elements in HDPE and PVC leachates and control media (AMP1) detected by ICP-OES and ICP-MS and their factor of increase in leachates relative to the control.

Method	Element	Unit	Method Detection Limit	Control (AMP1)	HDPE leachate	PVC leachate	HDPE factor of increase (leachate/control)	PVC factor of increase (leachate/control)
**ICP-OES**								
	Ca	mg/L	10	376.83±33.66	390.2±31.85	381.9±25.68	1.04	1.01
	Na	mg/L	20	11219.42±944.51	10900.51±983.9	10748.56±565.14	0.97	0.96
	S	mg/L	10	973.22±114.3	949.74±77.39	959.52±72.12	0.98	0.99
**ICP-MS**								
	Al	µg/L	5	36.46±7.8	83.77±33.01	31±3.53	2.30	0.00
	B	µg/L	5	34.82±12.03	39.1±11.98	45.36±10	1.12	1.30
	Ba	µg/L	5	19.26±7.3	23.78±7.1	29.14±7.42	1.24	1.51
	Cu	µg/L	5	1.53±2.4	3.07±3.15	5.92±1.76		
	Mn	µg/L	5	2.58±0.37	6.1±1.46	4.56±0.73		
	Ni	µg/L	5	4.32±0.68	6.81±2.2	4.69±0.74		
	Sr	µg/L	5	43.64±10.86	50.82±10	298.51±126.77	1.16	6.84
	Zn	µg/L	5	10.95±14.03	334.95±137.65	6179.45±2123.21	30.58	564.12

Concentrations lower than the method detection limit are shown in grey font. Factors of increase were not calculated where measurements were below the accurate detection limit for control media.

Si, As, Cd, Cr, Mo,Pb, Sn, V concentrations were under the detection limit for both leachate and control (values not shown).
