# Supplementary material for: Plastic leachates impair growth and oxygen production in Prochlorococcus, the ocean’s most abundant photosynthetic bacteria
Source: Commun Biol. 2019 May 14;2:184. doi: 10.1038/s42003-019-0410-x (PMC6517427; doi:10.1038/s42003-019-0410-x)
Supplement: Supplementary file 2 — Description of Additional Supplementary Files [file 42003_2019_410_MOESM2_ESM.pdf]

Description of additional supplementary items

Supplementary Dataset 1. (separate file)
This file contains the source data for Fig. 1 and results of statistical analyses of growth parameters conducted with one-way ANOVA followed by Tukey's multiple comparison tests.

Supplementary Dataset 2. (separate file)
This file contains the source data for Fig. 2 and results of statistical analyses of effective quantum yield of PSII (Φ_{PSII}) and oxygen production rates conducted with one-way ANOVA followed by Tukey's multiple comparison tests.

Supplementary Dataset 3. (separate file)
This file contains a list of the full set of *Prochlorococcus* MIT9312 genes for which significant differential expression was observed following short-term exposure to HDPE or PVC leachate (relative to AMP1 control).

Supplementary Dataset 4. (separate file)
This file contains a list of the full set of *Prochlorococcus* NATL2A genes for which significant differential expression was observed following short-term exposure to HDPE or PVC leachate (relative to AMP1 control).

Supplementary Dataset 5. (separate file)
This file contains the list of database matches for components strongly enriched in PVC and HDPE leachate (50 g/L plastic in AMP1) compared to corresponding control (AMP1) detected by LC-MS/MS.
.

Supplementary Dataset 6. (separate file)
This file provides the unfiltered peak data from the LC-MS analysis of AMP1, PVC and HDPE leachate chemistry
