# Supplementary material for: Plastic leachates impair growth and oxygen production in Prochlorococcus, the ocean’s most abundant photosynthetic bacteria
Source: Commun Biol. 2019 May 14;2:184. doi: 10.1038/s42003-019-0410-x (PMC6517427; doi:10.1038/s42003-019-0410-x)
Supplement: Supplementary file 3 — Reporting Summary [file 42003_2019_410_MOESM3_ESM.pdf]

nature research

Corresponding author(s): Dr. Sasha Tetu

Reporting Summary

Nature Research wishes to improve the reproducibility of the work that we publish. This form provides structure for consistency and transparency in reporting. For further information on Nature Research policies, see Authors & Referees and the Editorial Policy Checklist.

Statistical parameters

When statistical analyses are reported, confirm that the following items are present in the relevant location (e.g. figure legend, table legend, main text, or Methods section).

n/a	Confirmed	
☐	☒	The exact sample size (*n*) for each experimental group/condition, given as a discrete number and unit of measurement
☐	☒	An indication of whether measurements were taken from distinct samples or whether the same sample was measured repeatedly
☐	☒	The statistical test(s) used AND whether they are one- or two-sided<br>*Only common tests should be described solely by name; describe more complex techniques in the Methods section.*
☒	☐	A description of all covariates tested
☐	☒	A description of any assumptions or corrections, such as tests of normality and adjustment for multiple comparisons
☐	☒	A full description of the statistics including central tendency (e.g. means) or other basic estimates (e.g. regression coefficient) AND variation (e.g. standard deviation) or associated estimates of uncertainty (e.g. confidence intervals)
☐	☒	For null hypothesis testing, the test statistic (e.g. *F*, *t*, *r*) with confidence intervals, effect sizes, degrees of freedom and *P* value noted<br>*Give P values as exact values whenever suitable.*
☒	☐	For Bayesian analysis, information on the choice of priors and Markov chain Monte Carlo settings
☒	☐	For hierarchical and complex designs, identification of the appropriate level for tests and full reporting of outcomes
☒	☐	Estimates of effect sizes (e.g. Cohen's *d*, Pearson's *r*), indicating how they were calculated
☐	☒	Clearly defined error bars<br>*State explicitly what error bars represent (e.g. SD, SE, CI)*

Our web collection on statistics for biologists may be useful.

Software and code

Policy information about availability of computer code

Data collection	no software used
Data analysis	Progenesis QI (Waters), Compound Discoverer (ThermoFisher Scientific),CytExpert (Beckman Coulter), Prism 7 (GraphPad), EDGE-pro v1.31, subread v1.5.2, DESeq2 in R version 3.3.3.

For manuscripts utilizing custom algorithms or software that are central to the research but not yet described in published literature, software must be made available to editors/reviewers upon request. We strongly encourage code deposition in a community repository (e.g. GitHub). See the Nature Research guidelines for submitting code & software for further information.

Data

Policy information about availability of data

All manuscripts must include a data availability statement. This statement should provide the following information, where applicable:

- Accession codes, unique identifiers, or web links for publicly available datasets
- A list of figures that have associated raw data
- A description of any restrictions on data availability

All data associated with papers is available in main or supplementary tables and figures. Raw and analyzed transcriptomic data are accesible via NCBI GEO database, #GSE118155.

Field-specific reporting

Please select the best fit for your research. If you are not sure, read the appropriate sections before making your selection.

☒ Life sciences ☐ Behavioural & social sciences ☐ Ecological, evolutionary & environmental sciences

For a reference copy of the document with all sections, see nature.com/authors/policies/ReportingSummary-flat.pdf

Life sciences study design

All studies must disclose on these points even when the disclosure is negative.

Sample size	Three independent biological replicate cultures were acclimated and transfers from each were used as replicates in each experiment. Preliminary experiments indicated that this level of replication provided minimal variability between replicates allowing robust statistical analysis.
Data exclusions	Data was excluded for one oxygen measurement (NATL2A HDPE exposure 0% leachate control, 3 hr) due to data indicative of an air bubble in sample vial.
Replication	All experiments were performed with 3 independent biological replicates. Leachate exposure experiments were performed for each strain on 2-3 independent occasions each with 3 biological replicates, however only PAM and culture density (OD750) measurements were collected for preliminary experiments not reported in this manuscript. Assays were highly reproducible for all measurements collected.
Randomization	Three independent, random subcultures were selected to establish each biological replicate for use in each experiment.
Blinding	Investigators were not blinded to group allocation during data collection and analysis.

Reporting for specific materials, systems and methods

Materials & experimental systems

n/a	Involved in the study
☐	☒ Unique biological materials
☒	☐ Antibodies
☒	☐ Eukaryotic cell lines
☒	☐ Palaeontology
☒	☐ Animals and other organisms
☒	☐ Human research participants

Methods

n/a	Involved in the study
☒	☐ ChIP-seq
☐	☒ Flow cytometry
☒	☐ MRI-based neuroimaging

Unique biological materials

Policy information about availability of materials

Obtaining unique materials	There are no restrictions on material availability

Flow Cytometry

Plots

Confirm that:

☒ The axis labels state the marker and fluorochrome used (e.g. CD4-FITC).

☒ The axis scales are clearly visible. Include numbers along axes only for bottom left plot of group (a 'group' is an analysis of identical markers).

☒ All plots are contour plots with outliers or pseudocolor plots.

☒ A numerical value for number of cells or percentage (with statistics) is provided.

Methodology

Sample preparation	Samples were diluted (50 uL of cells into 450 uL sterile filtered AMP1 media), immediately fixed with paraformaldehyde (final

Sample preparation	concentration of 1%), incubated in the dark at 4C for 1 hour, then frozen in -80C until later analysis.
Instrument	CytoFLEX S flow cytometer with 3 lasers (488, 638 and 405 nm) (Beckman Coulter)
Software	CytExpert software v2.2 (Beckman Coulter)
Cell population abundance	Dilute, fixed culture aliquots were thawed in the dark and further diluted 1:10 in sterile filtered AMP1 media, then run in a 96-well plate. Samples were run with a flow rate of 10 uL/min with data collected for 90 sec (15 uL). Chlorophyll fluorescent cells with SSC properties indicative of Prochlorococcus cells were gated as shown in Fig S2.
Gating strategy	Acquisition threshold for sample collection was set at 500 for the 690/50 BP (Chlorophyll A) channel. Samples were gated with the Prochlorococcus gate illustrated in Fig S2 to collect counts from pigmented and intact cells. No staining was performed.

☒ Tick this box to confirm that a figure exemplifying the gating strategy is provided in the Supplementary Information.
